# The Relationship between Nature Deprivation and Individual Wellbeing across Urban Gradients under COVID-19

**DOI:** 10.3390/ijerph18041511

**Published:** 2021-02-05

**Authors:** Linda Powers Tomasso, Jie Yin, Jose Guillermo Cedeño Laurent, Jarvis T. Chen, Paul J. Catalano, John D. Spengler

**Affiliations:** 1 Department of Environmental Health, Harvard T.H. Chan School of Public Health, Boston, MA 02115, USA; jieyin@hsph.harvard.edu (J.Y.); jcedenol@hsph.harvard.edu (J.G.C.L.); spengler@hsph.harvard.edu (J.D.S.); 2 Population Health Sciences, Harvard University, Boston, MA 02115, USA; jarvis@hsph.harvard.edu; 3 College of Architecture and Urban Planning, Tongji University, Shanghai 200092, China; 4 Department of Social and Behavioral Sciences, Harvard T.H. Chan School of Public Health, Boston, MA 02115, USA; 5 Department of Data Science, Dana-Farber Cancer Institute, Boston, MA 02115, USA; catalano@hsph.harvard.edu; 6 Department of Biostatistics, Harvard T.H. Chan School of Public Health, Boston, MA 02115, USA

**Keywords:** nature exposure, nature deprivation, health disparities, wellbeing, built environment, urban health interventions

## Abstract

Lockdown aiming at slowing COVID-19 transmission has altered nature accessibility patterns, creating quasi-experimental conditions to assess if retracted nature contact and perceived nature deprivation influence physical and emotional wellbeing. We measure through on-line survey methods (*n* = 529) how pandemic mandates limiting personal movement and outdoor nature access within the United States affect self-assessed nature exposure, perceived nature deprivation, and subsequent flourishing as measured by the Harvard Flourishing Index. Results indicate that perceived nature deprivation strongly associates with local nature contact, time in nature, and access to municipal nature during the pandemic, after controlling for lockdown mandates, job status, household composition, and sociodemographic variables. Our hypothesis is that individuals with strong perceived nature deprivation under COVID-19 leads to diminished wellbeing proved true. Interaction models of flourishing showed positive modification of nature affinity with age and qualitative modification of nature deprivation with race. Our results demonstrate the potential of local nature contact to support individual wellbeing in a background context of emotional distress and social isolation, important in guiding public health policies beyond pandemics.

## 1. Introduction

### 1.1. Nature’s Beneficial Impact on Health

Evidence of nature’s beneficial impact on physical [1,2,3,4,5], cognitive [6,7,8,9], and emotional health [10,11,12,13] is well substantiated in scientific literature. Nearly all studies conclude that health outcomes improve with exposure to non-threatening outdoor nature; the few studies which examine indoor nature exposure mostly yield positive associations [14,15,16,17]. Variability of nature exposure differentiates one’s experience in nature as well as response to nature contact, such that health outcomes are influenced by appropriate “dose” [18,19], frequency of contact [20,21], quality of nature exposure [22,23], biodiversity level [24,25,26,27], aesthetic preference [28,29], and urban greenspace proximity [30,31]. Even with emerging awareness of these distinctions, Kuo [32] infers that cumulative exposure to green *in toto*—parcel size, nature type, vicinity, etc.—is responsible for imparting nature’s health benefits.

Studies of nature–health relationships often approach exposure additively. Intervention studies [33,34,35,36] frequently contrast participant response in non-natured built environments versus nature-dense urban parks. Observational studies [4,37,38,39,40,41] analyze proximity and expanse of residential greenspace in increasing increments using spatial measures such as Normalized Difference Vegetation Index (NDVI), a satellite-based capture of vegetation based on photosynthetic reflectance, or locational data such as GPS. Epidemiological methods [42,43,44,45] have identified a range of health improvements, e.g., lower morbidity and annual disease prevalence, applying these tools. These studies nonetheless lack data on individual nature use and specific participant characteristics, making it difficult to learn which exposure factors most strongly impact outcome effects. A reverse scenario of nature deprivation or withdrawal from nature has rarely been empirically explored owing to scenario improbability, the ethics of withdrawing salutogenic stimuli in experiments involving humans, and from a pragmatic study design point, leakage within non-exposed groups. The extraordinary situation of a global health crisis, compulsory shelter-at-home policies, and changes in the supply of and demand for outdoor nature areas has shaped conditions for a natural experiment in which to study how alterations in established patterns of nature exposure may affect individual wellbeing under a state of generalized societal adversity. This paper associates changes to nature contact under COVID-19 with individual wellbeing.

### 1.2. Nature Deprivation

COVID-19′s emergence in late 2019 occasioned urgent public health issuances of lockdown mandates and suspended daily activity patterns including nature-seeking throughout much of the world. In the United States, lockdown or “shelter-in-place” protocols mandated closures of school, travel, and non-essential businesses in mid-March 2020 [46]. The lengthy period of home confinement imposed in many U.S. cities and states, as well as concomitant restrictions on local nature access, has focused public health concerns on changes to individual wellbeing, a construct summating positive emotion, engagement, relationship, meaning, and achievement [47]. From a wellbeing perspective, many individuals go outdoors seeking physical activity, socialization, and emotional resilience derived from immersion in natural environments. Green exercise, i.e., physical activity performed outdoors, offers known remediation pathways for wellbeing improvements [48,49,50,51]. Because immersion in nature has been shown to mitigate feelings of anxiety [52] and attention deficit [53], and promote self-efficacy [54,55] and meaningfulness [56], habitual nature contact may provide an essential coping mechanism for many people during times of distress. Current lockdown policies may be causing nature-dependent individuals to experience feelings of nature deprivation in situations where habitual nature-seeking behaviors might otherwise have assuaged a heightened pathogenesis brought on by health, financial, or emotional concerns stemming from the COVID-19 pandemic [57].

While we do not presume that most individuals experienced total separation from nature under COVID-19, the overlay of restricted personal mobility and closure of public nature sites like state and national parks—what nature-seekers refer to as “the nature I desire” [58]—irrefutably contracted the supply of nature and individual access to it. Furthermore, lockdown restrictions in many states and urban cities have created immediate and captive audiences for nature where it is circumstantially found. In densely built environments, publicly managed greenspace governs opportunities for nature exposure, unlike less densely developed areas with private greenspace or open viewsheds. Restrictions on public greenspace—urban vegetation for recreational use [59]—and blue space—outdoor water environments [60]—therefore may directly relate to wellbeing, particularly within urban settings.

### 1.3. Background Literature

Multiple studies indicate that exposure to nature amplifies beneficial physical, psychological, and emotional outcomes. Nature comprehends “the range of scale and degrees of human management, together with […] sunsets and mountain views”, as suggested by Frumkin et al. [1]. Nature connectivity is core to the environmental psychology literature regarding pro-environmental attitudes and behaviors [61,62,63,64]. Measures which operationalize the concept of nature affinity or connectivity share a common construct [65,66] rooted in positive affect, though their predictive power does not extend to the effects of nature withdrawal. Nature connectivity has been positively linked with psychological resilience and maintenance of positive mental health under challenge [67] and individual reliance on favorite places in nature for restoration [29]. However, as far as we know, only one study [68] formally analyzes nature connectivity as a potential modifier of nature contact to assess wellbeing and pro-environmental behaviors.

Our study considers both nature connectivity and the lesser-explored feelings of nature deprivation under conditions where altered nature contact may be perceived to insufficiently support challenges to individual wellbeing. The selected outcome of flourishing amplifies the notion of human wellbeing to include physical and emotional health, virtue, and adequate material sustenance [69]. Because nature exposure has been linked to sentiments of positive psychological functioning [26,64], flourishing is a suitable outcome to measure the impact of nature deprivation under conditions of psychological challenge and adversity. Although prior studies [70,71] have empirically associated wellbeing with nature forms, our study is also the first to apply the Harvard Flourishing Index as a formal psychometric construct to relate perceived nature deprivation to flourishing. Harvard’s Human Flourishing Program provides an empirical framework for integrating quantitative social sciences methods across disciplines to better understand the health implications of human flourishing.

Many studies [72,73,74,75] have considered the association of green space in one’s immediate residential vicinity to health outcomes. As urbanization displaces traditional venues for nature contact, nearby greenspace increasingly becomes a key exposure criterion for health outcomes. Some researchers theorize [76,77] that individuals with lower mobility—the elderly and children—and those of lower socioeconomic status (SES) concentrate their outdoor activities closer to home. Mental illness and emotional disorders strongly relate to proximate green space [18,78,79]. Improved emotional wellbeing and mental health outcomes that derive from increased nature exposure have shown further to preferentially benefit residents with less neighborhood greenspace, as often typical of low SES areas [80,81,82]. Nature found in the immediate neighborhood vicinity thus takes on a larger predictive role to accommodate intentional outdoor nature-seeking in the absence of routinely accessible natural sites.

Loss of nature contact has been widely reported [32,83,84] and attributed to urban lifestyles, with ensuing disengagement from and disaffection for nature repercussive for human and planetary health. Researchers have measured baseline indicators of time in nature to conclude that absence of nature contact is the population norm, with the consequence of “nature deprivation” [85] as permanent removal of nature contact being vulnerability to a range of negative health outcomes. Shared concern for a phenomenon described as the “rarity of direct experience in nature” [86] and characterized in the literature as nature deficiency [87] or nature impoverishment [23] has led to research on prescriptive nature re-engagement [88,89] and nature-based health treatment [90], especially among children. Still, conceptualization of nature deficit disorder [91] remains a descriptive and not diagnostic condition, with formal study elusive given research bounds, though a few exceptions exist [92].

The term nature deprivation here denotes perceived nature deficiency, i.e., unmet personal need to access the nature one desires, rather than comparative resource inadequacy described in relative deprivation theory, where individuals or groups cognitively appraise their situation as unfairly disadvantaged [93,94]. We draw attention to our use of this term in the emotional sense of withdrawal from habituated forms of nature exposure rather than as the relative area-level absence of green or blue space. To our knowledge, ours is the first study to examine the effects on wellbeing from diminished or withdrawn nature exposure, particularly where society-wide restrictions on personal mobility and the accessibility of some types of nature-rich areas account for nature separation.

### 1.4. Hypothesis and Purpose

In this study, we hypothesize that changes to nature exposure at the indoor, neighborhood, and municipal levels will induce feelings of individual nature deprivation during the period of COVID-19 restrictions, adjusting for age, gender, race, urbanicity, area-level poverty, and US geographic region, as these variables may confound the nature exposure–nature deprivation relationship [95]. Second, we posit that individuals who feel deprived of nature will experience a loss of baseline flourishing after accounting for job status and household composition under the pandemic. While our exposure of interest consists of nature contact potentially modified by pandemic restrictions, we hypothesize that subjective nature affinity may underlie pre-established patterns of nature pursuit that will continue under sheltering. We additionally consider secondary objectives specific to restrictive policies existing at the time of survey-taking, given information available from our results. First, will municipal restrictions on publicly managed nature areas influence feelings of nature deprivation under lockdown? Second, will pre-existing patterns of nature exposure impact subsequent wellbeing when habitual nature contact is altered? Third, do sociodemographic variables modify relationships with self-defined nature affinity and self-expressed nature deprivation that predict individual flourishing during COVID-19-like conditions?

## 2. Materials and Methods

### 2.1. Survey Population

Our study population consisted of individuals originally recruited for focus group interviews to explore formative experiences and origins of attitudes shaping nature-seeking behaviors as adults. Study participants were recruited and directed to an on-line enrollment portal through Facebook advertisements placed October 2019 in four regionally distinct metropolitan areas representative of differing climate, topography, and urban building density—Boston, Atlanta, San Francisco, and Phoenix. Additional focus group recruitment occurred with the assistance of university faculty in the targeted metropolitan areas in the attempt to diversify the age, race, and gender of the study base. The original study base (*n* = 625) had already voluntarily enrolled and consented through electronic recruitment. We had no pre-existing data on study participants.

A new recruitment email for this study addressing exposure to nature under COVID-19 restrictions was sent to all enrolled study participants via email addresses on file explaining survey study objectives and linking to a Qualtrics-distributed on-line survey instrument. This survey is found in Supplementary Materials S1. Study participants were sent a reminder email at seven and 14 days to request survey completion. No contact was made with study participants after two attempts. A survey link specific to the enrollment ID of each originally enrolled participant allowed us to monitor response rates of the initial cohort at 37.6%; a second, non-specific survey link created for survey forwarding allowed us to track the snowball effect of the study design that provided 62.4% of our study population. Survey forwarding extended the initial four metropolitan areas to 36 U.S. states, Puerto Rico, and the District of Colombia.

Six-hundred participants returned the survey during the month the link was active. Survey-takers who omitted items were dropped from the study, resulting in a final sample size of 529 participants. This final study population resided in areas of population density (large urban areas and suburbs each represented 36% of respondents), were majority female (75%), white non-Hispanic (82%), and of slightly younger age (age 25–34 = 29%) although no age category was below 10.6%. Individuals who identified as black or Hispanic lived within zip codes of on-average lower NDVI levels and higher poverty rates. Nature affinity scores rose with age, consistent with findings of higher nature connectedness at progressive age [96,97,98]. The Harvard T.H. Chan School of Public Health Institutional Review Board approved both the original focus group and survey studies under IRB 19-1419 on 21 April 2020.

### 2.2. Outcome Measures

Our main outcomes of interest were individual feelings of nature deprivation, operationalized across five levels of agreement with the statement, “I feel nature deprived since coronavirus restrictions were imposed”, and subsequent flourishing self-assessed through survey items comprising the Harvard Flourishing Index. The exploratory nature of this measure meant we approached the development of nature deprivation in terms of content validity, operationalizing the concept under the domains of behavior (time in nature), affect (sense of withdrawal, isolation), cognition (restlessness, loss of temporal awareness), somatic symptoms (vitality, lethargy), and motivation (goal-abandonment). The Harvard Flourishing Index (HFI), a validated measurement approach to human flourishing [99], highlights five central domains nearly universally apprised as vital elements of human wellbeing: happiness and life satisfaction, mental and physical health, meaning and purpose, character and virtue, and close social relationships; a sample item from Domain 4 is “I always act to promote good in all circumstances, even in difficult and challenging situations.” Supplementary Materials S2, contains the complete HFI. This Index was selected over alternative subjective wellbeing indices for its optional sixth domain, financial and material stability, deemed highly relevant to COVID-19′s implications of wage or job loss on individual wellbeing within the United States. Each of the flourishing questions was assessed on a 10-point scale, with flourishing outcomes calculated on a continuous 0–90 scale.

We substituted resilience measures for the Flourishing Index’s two happiness items due to potential temporal confounding as pandemic malaise. Happiness has previously been positively associated with nature exposure [70,96,100], though the COVID-19 Response Tracking Study conducted 21–29 May 2020 (*n* = 2279 adults nationwide) and the General Social Survey reported the highest percentage of individuals since record-keeping began in 1972 (23%) responding at the lowest level of happiness [101]. A third scale item, “My relationships are as satisfying as I would want them to be”, similarly was measured in terms of resiliency as “I feel close to others in my community.” Cronbach’s alpha test of internal consistency was performed on the nine remaining items comprising the Flourishing Index, resulting in an alpha of 0.82, a high indicator of composite scale reliability.

We tested for missingness and dropped survey responses containing omitted values. We did not adjust for length of lockdown due to response homogeneity: 85.97% of respondents were under restriction for 4 weeks, 6.85% for 3–4 weeks, and 5.87% considered essential workers temporally unaffected by restrictions. A second covariate, mode of transportation, was dropped from analysis due to similar response homogeneity, with under 1% using public transit, a marker initially presumed to associate with accessing nature under COVID-19.

### 2.3. Exposure

Our exposure of interest was cumulative nature contact experienced by individuals during lockdown restrictions at the time of survey-taking. We analyzed three levels of nature exposure—indoor, neighborhood, and municipal—under conditions of pandemic restrictions. We distinguished in situ, i.e., incidental, nature contact from intentional nature desired for outdoor activities [102]. Survey-takers cited intentional nature-based activities, e.g., hiking, kayaking, and community gardening, as most commonly missed due to pandemic restricted non-accessibility.

**Figure 1:**
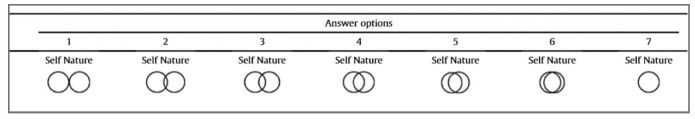
Inclusion of nature in self-scale (Schultz, 2002) used in survey to indicate self-expressed nature affinity.

The term “outdoors” was explicitly defined for survey-takers as “time intentionally spent in or near nature: backyard, outdoor gardening, urban park, other urban/suburban greenspace, greenway for walking or biking, open woodlands, state or national park/forest/seashore, all forms of outdoor sport or recreation”. Indoor nature exposure was represented by four measures: nature seen through window views, stage of springtime nature reemergence, having indoor plants, and having a pet. Neighborhood-level exposure consisted of summative nature contact dictated by local shelter-in-place policies (four categories: complete lockdown; can go outside but do not come in contact with nature; can go outside but preferred nature is inaccessible; and no pandemic restrictions: normal outdoor access) and by a second item comparing amount of time spent outdoors in local nature under COVID-19 vis-à-vis pre-pandemic conditions (three categories: less, same, and more). Municipal-level nature exposure concerned access to municipally-managed nature areas such as parks, conservation lands, and beaches (four categories: full access with social distancing required, reduced parking to limit park occupancy, non-vehicular foot or bicycle egress only into parks, and complete closure of nature areas to the public). Frequency of time in nature prior to COVID-19 restrictions (four categories: never or <monthly; 1–2x/month; 1–2x/week; 3–4x/week; daily) provided a baseline measure of nature exposure and was used as a variable to predict nature deprivation. Nature affinity was self-assessed through the single-item measure Inclusion of Nature in Self (INS) [103] addressing degree of connectedness with nature, where option (1) represents no overlap between nature and self, and option (7) represents oneness with nature (Figure 1). Because distribution of INS responses was positively skewed, scores were transformed from a seven-point continuous scale into a three-point categorical indicator by collapsing levels one through three as Low Nature Affinity, levels four and five as Medium Affinity, and levels six and seven as High Nature Affinity. Nature affinity was tested both as an independent variable as well as a modifier of sociodemographic variables to predict flourishing.

We tested for collinearity between nature affinity and nature deprivation to ensure these two variables independently predict flourishing outcome values. A Spearman correlation test of the relationship between nature affinity and feeling nature deprived produced a non-significant −0.002 correlation.

### 2.4. Covariates

Individual-level covariates assessed in this survey were age (18–24 = reference, 25–34, 35–44, 45–54, 55–64, 65–74, and ≥75), gender (male = reference, female, non-binary), urbanicity (large city = reference, suburbs, small town, and rural area), race (Non-Hispanic White = reference, Native American or Alaska Native, Black or African American, Hispanic or Latinx, Middle Eastern, Native Hawaiian or Pacific Islander, South or East Asian), and zip code in which the survey was taken. Decadal age was analyzed as a five-category covariate after merging the upper three categories—55–64, 65–74, and ≥75—into a single upper-tier category of over-55 age based on statistically similar modeling output. Race was analyzed dichotomously (Non-Hispanic white vs. Non-white) due to the overrepresentation of white (81.94%) and underpowered seven non-white response categories.

Five-digit zip codes provided by respondents were cross-referenced with U.S. Census Zip Code Tabulation Area data on percent of population living under the poverty line from The Public Health Disparities Geocoding Project and with urban population density to control for socio-economic variation which might relate to neighborhood-level nature exposure [104]. We chose to measure geo-coded area-level SES positions rather than individual SES position given the study’s potential to appear as an extension of research sponsorship. Area-level poverty was categorized as low = reference (0.0–0.05%), low-medium (0.05–0.10%), medium (0.10–0.20%), and high (0.20–1.0) with ≥96 entries in each category. We also assessed current employment status (five categories: not working, e.g., retired = reference; working virtually or from home; working in a position deemed “essential”; some loss of wages due to coronavirus; and loss of job due to workplace closure) and household composition (five categories: live alone = reference; live with spouse or partner; live with parents; have children under the age of 18 living at home with me; share living space with roommates) as outcome-related predictors of flourishing since coronavirus measures were imposed.

### 2.5. Analytical Approach

We used bivariable and multivariable regression analysis in this study to predict associations of levels of nature exposure to individual flourishing under lockdown restrictions. Each nature exposure variable was examined through bivariate correlation to derive statistical significance at the individual test level and to screen as multi-degree of freedom test based on F-statistics and *p* values. This determined which variables produced meaningful effect estimates of nature deprivation and flourishing. We conceptualized our model building behind the signal strength of individual exposure variables found to describe our principal outcome and, from those descriptions of bivariable significance and effect, constructed our final multivariable exposure models. Nature exposure factors associating significantly in bivariable modeling of flourishing were retained. Multi-degree of freedom tests showing no statistical significance resulted in the remaining exposure items being eliminated from the full multivariable models of nature deprivation. Bivariable models examining confounding by sociodemographic variables showed only age, race, and urbanicity to relate both to exposure factors and nature deprivation, though we accounted for all six sociodemographic variables, adding gender, area-level poverty, and region in this model. Measures of association are reported as unstandardized betas, 95% confidence intervals and *p*-values.

Extensive consideration was given to how moderation of our subjective variables by sociodemographic differences may associate with wellbeing under COVID-19 and the potential limitations this may impose on exposure variables. We assessed effect modification by factor levels of age, gender, race, urbanicity, area-level poverty, and region on categorical nature affinity and perceived nature deprivation. We also evaluated the statistical strength of interaction terms by using nested F-tests for linear regression models. All statistical analyses were performed using R version 3.6.3, and models were run using package ‘gamm4′, version 0.2-5 [105].

## 3. Results

### 3.1. Main Findings: Bivariate and Multivariate Models

Bivariable and multivariable regression of indoor, neighborhood, and municipal level nature exposures consistently predicted changes to individual nature deprivation under the pandemic, after adjusting for sociodemographic factors of age, gender, race, urbanicity, area-level poverty, and region as well as baseline nature affinity. Table 1 provides a demographic breakdown of the final study population stratified by response levels of perceived nature deprivation.

Table 2 shows medium and high levels of nature affinity strongly predicted higher perceived nature deprivation. Greater time spent in nature under COVID-19 restrictions vis-à-vis pre-pandemic had the most pronounced effect on reducing feelings of deprivation among all exposure variables (−1.07, 95% CI: (−1.32, −0.81), *p* ≤0.001). Other exposure variables significantly associated with lower perceived nature deprivation were public nature parks and reserves remaining fully open under the pandemic as compared to restricted entry policies, attenuated COVID-19 sheltering policies, pet ownership, older age, and Western U.S residence. Female gender was the only sociodemographic variable to associate positively and significantly with nature deprivation.

Table 3 assesses the relationship between nature deprivation and flourishing. Feeling nature-deprived produced strong effect signals at high levels of significance in both bivariable and multivariable models. The item assessing perceived nature deprivation effectively asked survey-takers if their nature contact under quarantine met their needs. Survey-takers who strongly agreed with the statement, “I feel nature deprived under coronavirus restrictions,” showed a significant flourishing decline of 4.04 units (95% CI: (−7.33, −0.74), *p* = 0.02) relative to those who strongly disagreed with feeling nature deprived under a multivariable model. Individuals who agreed with feeling deprived lost 2.72 flourishing units at a marginal level of significance (95% CI: −5.68, 0.24, *p* = 0.07). Those who neither agreed nor disagreed with feeling nature deprived still exhibited a non-significant loss of flourishing compared to those not feeling deprived of nature. Bivariable modeling of strongly feeling nature deprived similarly predicted high flourishing losses (−5.52, 95% CI: (−8.80, −2.25), *p* ≤0.001), an effect exceeding all factors except job loss due to the pandemic (−7.54, 95% CI: (−11.62, −3.45), *p* ≤0.001).

### 3.2. Main Findings: Interaction Models

**Figure 2:**
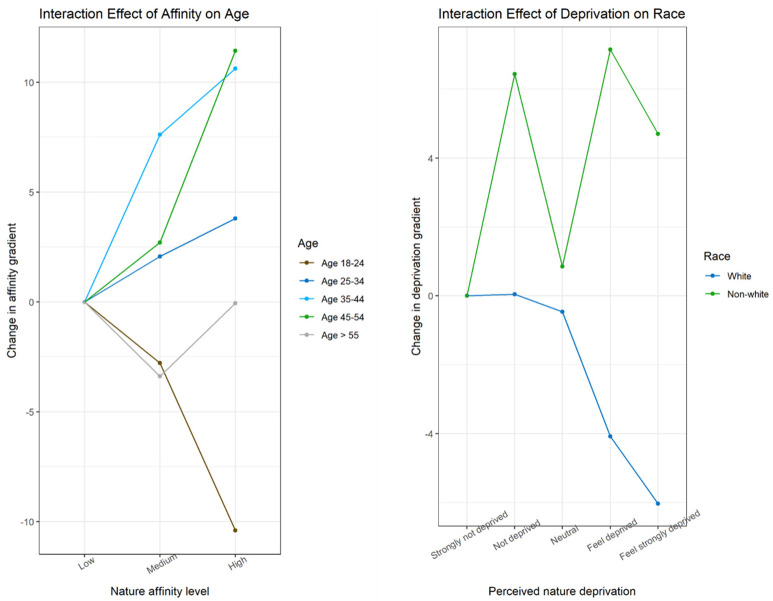
Interaction models of nature affinity and age (**left**) and nature deprivation and race (**right**) on flourishing. (**Left**) Stronger affinity associated with flourishing loss in ages 18–24 but gains in mid-age groups. (**Right**) Stronger perceived deprivation related to flourishing losses among whites but gains among non-whites.

Our modification models showed affinity interacted positively with the 35–54 age range categories but negatively with the youngest (18–24) and oldest (over 55) categories in predicting flourishing (Figure 2, left). Nature deprivation modified race, with white non-Hispanic individuals experiencing negative effects on flourishing outcomes and non-white individuals showing positive effects on flourishing (Figure 2, right). Net interaction effects in Table 4 measures how affinity varies among individuals of a given age category, such that medium affinity among individuals age 25–34 = −2.78 (medium affinity) + 4.85 (interaction effect) = 2.07. No other potential modifier proved statistically significant in our models.

## 4. Discussion

### 4.1. Summary of Results

Our main findings support both our hypotheses that (1) changes to nature exposure identified at the indoor, neighborhood, and municipal levels related to self-expressed nature deprivation under the COVID-19 pandemic, with those who spent the same or more time in nature under shelter-in-place feeling significantly less deprived after accounting for sociodemographic variables and stringency of pandemic restrictions on personal mobility and public nature access; and (2) individuals who felt nature deprived under shelter-in-place experienced reduced flourishing at high statistical significance, controlling for job and household factors. These results were analyzed within strata of nature exposure and independently of background declines of flourishing during COVID-19 found in national online sampling [106].

#### 4.1.1. Redistributed Time in Nature under Lockdown

Our survey data show a slight overall decline but a large redistribution in the amount of time individuals spent outdoors under COVID-19, not unexpected given business closures and state and municipal limits on non-essential activity. One-hundred-and-fifty-one individuals (23.3%) spent much less time outdoors, 122 (18.8%) less time, 132 (20.4%) the same time, 147 (22.7%) more time, and 96 (14.8%) much more time. Declines in flourishing corresponded monotonically to individual feelings of nature deprivation since shelter-in-place began. It is possible that transition to virtual or reduced work and shuttered alternate activities enabled some individuals to experience greater nature contact, though others were unable to access desired nature venues for context-specific outdoor activities. While we do not know how frequently those spending the same amount of time in nature went outdoors, individuals who reported feeling nature-deprived or strongly nature-deprived since sheltering began experienced a statistically significant drop in flourishing of 4.6% and 6.8%, respectively, compared to our reference group who strongly disagreed with feeling nature deprived. In comparison, pandemic-related wage loss and job loss were associated with a 5.5% and an 11.4% respective flourishing decline, compared to the reference group.

#### 4.1.2. Neighborhood Level Nature Contact

We found that neighborhood-level nature contact drove cumulative exposure under the pandemic, adding to previous evidence highlighting the relevance of neighborhood greenspace in health outcomes. COVID-19 shelter-at-home policies restricting personal mobility, destination availability, and openness of public spaces preclude or constrict choice of nature contact for many people, leaving incidental contact with indoor and neighborhood nature the de facto exposure. Intentional nature-seeking lapses to circumstantial contact with neighborhood-level tree-cover or greenspace availability under sheltering conditions. Interventions to re-introduce nature into urban areas should thus prioritize the greening of neighborhoods in ways that recognize highly localized, incidental contact with nature.

#### 4.1.3. Subjective Factors Relating Nature Contact to Pandemic Circumstance

While objective variables summarily captured cumulative nature exposure measures, subjective nature affinity and perceptual aspects of nature contact affected by COVID-19 additionally impacted feeling nature deprived. The item “How have coronavirus restrictions where you currently live affected your interaction with nature?” revealed that individuals who went outside but found their preferred nature inaccessible felt sixty percent more deprived of nature (−0.36, 95% CI: (−0.80, 0.07), *p* = 0.10) than those unaffected by lockdown. Likewise, feelings of nature deprivation rose commensurate with extent of restrictions imposed by local governments and nonprofit land trusts on natural areas considered high quality. “Given restrictions on personal mobility within your city or state, can you freely access nature areas such as parks, conservation land, beaches, etc.?” Perceived deprivation declined 3.2-fold among individuals where municipal nature sites remained completely open to the public as compared to parks having limits or bans on vehicle access (−1.79, 95% CI: (−2.24, −1.33), *p* ≤ 0.001). Stronger nature deprivation connected to closure or restricted park access in our study inversely mirrored greater levels of restoration experienced by participants in a previous study after visiting sites of higher environmental quality as compared with urban spaces [107]. Higher quality sites in that study were operationalized by protected/designated area status such as nature reserves, rural, and coastal locations, the same as those affected by pandemic restrictions or closures here.

Participants self-reported the amount of time spent in nature at two time points: prior to and under lockdown. Evaluating subjective nature deprivation subsequent to items around time in nature helped validate the deprivation construct through this retrievable information. Still, the use of subjective judgment to gauge the extent of nature deprivation might put in doubt the integrity of our findings. Self-reports of nature deprivation amidst the anomaly of lockdown offer no precedent to assess its quality or reliability, underscoring the challenge of measuring an invisible concept which lacks some correlated criterion validity. Greifeneder et al. suggest that researchers’ reliance on feelings as is not a “necessarily flawed heuristic” to be easily dismissed but a judgment strategy which in the main serves to efficiently carry information of generally stable validity [108]. Problems of measure validity here might still manifest as the over-reporting of nature deprivation as surrogate for other pandemic-related losses, as lockdown definitionally conveys a loss of freedom under which perceived nature deprivation becomes psychologically nested.

#### 4.1.4. Effect Modification by Socio-Demographics

Having expected nature affinity to be an informative qualitative indicator of feelings of nature deprivation, our tests for effect modification of sociodemographic variables on flourishing by the subjective variables of nature affinity and nature deprivation proved insignificant, but for two exceptions. Interaction between nature affinity was observed only for age, with strongest effects and significance among individuals ages 25 through 45. Our significant finding that affinity and flourishing vary with age contrasts with no effect moderation found previously for age but upholds an absence of significant effect for females [70]. Nature deprivation interacted negatively with white race yet positively with non-white race in determining flourishing outcomes. Additional analysis was then performed where we controlled for items which might explain the divergent flourishing results between the two racial categories. Non-whites exhibited much higher social support, which tended to increase flourishing at a level of positive effect equal to that lost to nature deprivation. Non-whites were also more likely than whites to be essential workers (e.g., health care, essential retail, operations and maintenance), a group which exhibited lower reductions in flourishing than individuals who had lost wages or jobs to COVID. While other outcome related variations may have differentially contributed to flourishing among white vs. non-white individuals, we believe the qualitative interaction that switches directions by race in part owes to our grouping of non-white races into a single binary category to achieve statistical power. The non-white category therefore assumes economic as well as racial heterogeneity, as well as other possible unmeasured confounding.

### 4.2. Policy Implications

Research on restricted access to nature during a period of high global distress is unique and timely. Our survey data captured across regional, socio-demographic, and density spectra call attention to individual reliance on nature for wellbeing and the detriments of restricting nature contact when its need is most acute. Prior evidence indicates that nature exposure stimulates salutogenic response pathways counteractive to negative emotional impulses [109,110]. This study further suggests that pre-existing patterns of nature-seeking may influence behaviors that reduce feeling nature deprived and support higher flourishing under lockdown. Understanding if nature exposure serves as a positive coping mechanism to buffer negative affect may inform long-term preparedness for population-level, environmentally triggered health emergencies well beyond disease. These include heat- and climate-related confinement and indoor retreat from hazardous air pollution incidents. Shelter-in-place is already common in Chinese and Indian cities during high PM2.5 occurrences [111,112]. These recurring episodes forecast the need for preventative wellbeing measures when indoor lockdown is required.

Public health policy recommendations pertinent to these research findings are fourfold: (1) decentralized and more equitable distributed urban greenspace, (2) demand management at public nature-based venues, (3) public health advisories on green exercise as an essential activity under specific sheltering conditions, and (4) investment in urban infrastructure to facilitate outdoor physical activity.

First, our study results show that shelter-in-place shifts the locus of urban nature contact from large parks to the neighborhood level. This shift results from closing or restricting large nature-based destinations, fear of taking public transit to reach distant greenspace, and displacement of recreational greenspace to accommodate large-scale COVID-19 testing sites like New York’s Central Park or San Francisco’s Golden Gate Park. The signal importance of neighborhood-level nature exposure which emerges reaffirms that urban landscapes must contain smaller, decentralized greenspaces accessible by walking.

Second, closure of public nature spaces diminishes wellbeing already eroded by indoor confinement, social isolation, and fear of disease transmission. Demand management and distributed access to public nature areas is preferable to total shuttering of nature spaces prone to transmit disease through overcrowding. Alphabetical assignment to certain days of the week or odd–even license plate park entry are already used by municipalities to regulate public access. Assisted reservation-based systems such as that used by the US National Forest and Park Services successfully manage public demand for nature and wilderness.

Third, public health guidance issued under San Francisco’s shelter-in-place order explicitly lists engagement in “outdoor activity, such as walking, hiking, or running, provided maintenance of social distancing” as an exempted essential activity under COVID-19 [113]. Cities can promote green exercise as a safe, preventative health behavior given appropriate precautions.

Fourth, government investment in neighborhood pedestrian green infrastructure can help to address on-going health risks associated with physical inactivity and social isolation further exacerbated by lockdown. Our results show that higher frequency of outdoor nature-seeking established prior to COVID-19 may have contributed to flourishing once pandemic restrictions were introduced. Subpopulations currently unable to engage in safe outdoor activity due to shortage of proximate urban infrastructure thus enter lockdown conditions already disadvantaged in terms of beneficial green exercise pattern-forming.

### 4.3. Study Limitations and Strengths

#### 4.3.1. Limitations

We acknowledge several limitations in this study which prevent us from drawing stronger conclusions or from generalizing to subpopulations underrepresented in the study base we analyzed. Among these are a relatively small study population; the lack of individual-level income or education variables, detail on the amount of time spent in nature and type of natural environments frequented during lockdown; cross-sectional design; and foremost, the subjectiveness of our central measure, perceived nature deprivation. No validated measurement scale exists for nature deprivation as it does for nature affinity or connectedness, leaving a blunt self-assessment of nature withdrawal as a one-item perceptual judgment.

Self-selection into the original focus group study may have attracted individuals inclined to report stronger nature-seeking attitudes and behaviors as compared to a general population, thus over-representing perceived nature deprivation under COVID-19. Because our original study recruitment targeted four large metropolitan areas with higher population density and distance to large-scale outdoor nature, the impact of restricted municipal nature access may appear more acute within this study population than for the U.S. population as a whole. More fundamental limits to result generalizability stem from a predominantly white (82%) and female (75%) study base. Despite targeted efforts to diversify our original study enrollment to include more male and non-white respondents, the snowball nature of survey link forwarding may have compounded the original imbalance of study population demographics.

Under-representation of some racial and ethnic groups gave us insufficient statistical power to stratify further on race despite eight options posed in our survey. Race was possibly confounded by unsettling social stigmatization associating Asian-identifying individuals related to COVID-19′s geographic origins in China [114,115]. While we controlled for race and urbanicity at the individual level, we modeled area-level SES, so it is likely that there is some residual confounding by race and income. Analysis of an income variable matched to respondent zip code and de facto urban vegetation levels extracted from high-resolution NDVI data could reduce the confounding potential by SES in associating nature exposure and wellbeing.

#### 4.3.2. Potential for Reverse Causality

Study results must be interpreted in light of the cross-sectional study design, precluding us from drawing any conclusions other than associative. Background severity of circumstances created by the COVID-19 pandemic admittedly implies that our analysis of survey data may be subject to other unmeasured individual confounders not assessed here and which might surface in a survey repeated over time. Implicit in any nature exposure research also is the question of bidirectionality: do people choose to live in greener areas due to high nature affinity, or does vicinity of greenspace cultivate engagement? The potential for reverse causality exists, particularly among individuals disinclined or unable to frequent outdoor nature for purposes of health or emotional restoration. Moreover, nature deprivation is just one of many means by which individuals may feel dispossessed under the COVID-19 pandemic; social, financial, and emotional deprivation also accompany sheltering.

#### 4.3.3. Strengths

This study provides preliminary evidence of how altered nature exposure may induce feelings of nature deprivation among individuals confronting the physical and emotional isolation of health restrictions, with diminished wellbeing prospects. It reaffirms the potential for mitigating adverse emotional health outcomes through prudent access to outdoor nature. Taking advantage of the widespread behavioral changes brought on by the COVID-19 pandemic for study design purposes reduced or removed many confounders implicit in measuring cumulative nature exposure, e.g., socialization to mediate nature exposure benefits [48]. Our survey data collection from 22 April to mid-May 2020 measured wellbeing one month into the pandemic but before large-scale urban protests around racial injustice broke out as a potential confounder of wellbeing among city dwellers. Finally, our collection of individual-level exposure data in this study, e.g., windows views, differences in seasonality and landscape, and work patterns, may inform further epidemiological study for assessing the accuracy of NDVI as an ecological-level proxy for nature exposure.

## 5. Conclusions

Substantial evidence exists for enhanced human health outcomes in the presence of nature. This survey study has shown that under widespread emergency policies of shelter-in-place, feelings of nature deprivation link strongly to individual wellbeing outcomes. Higher levels of cumulative nature exposure demonstrated lower nature deprivation scores as well as the reverse effect, that withdrawal of nature exposure and opportunities to pursue activity in nature compromise individual emotional health and wellbeing. Nature exposure was shown to offset reductions in nature exposure under quarantine for purposes of preventing human disease transmission. Lower nature deprivation was associated with higher flourishing. Policies that allow for the continuation and even increases in local nature contact should be part of public health strategies favoring precautionary shelter-in-place or lockdown under pandemics. Nature contact offers a means to proactively confront the emotional and physical health consequences corollary to social isolation and physical inactivity that COVID-19 has exposed.

## Figures and Tables

**Table 1:** Characteristics of the survey study population (N (% total)). *p* values indicate level of significance for a univariate test for differences in each variable across strata.

	Stratified by Perceptions of Nature Deprivation					
	Strongly Disagree That Feel Deprived	Disagreeon Feeling Deprived	Neither Agree nor Disagree That Deprived	Agree That Feel Nature Deprived	Strongly Agree That Feel Deprived	*p*
N = 529	93	82	71	169	114	
Nature Affinity (%)						0.62
Low Affinity	15 (16.1)	10 (12.2)	9 (12.7)	18 (10.7)	11 (9.6)	
Medium Affinity	43 (46.2)	44 (53.7)	36 (50.7)	96 (56.8)	54 (47.4)	
High Affinity	35 (37.6)	28 (34.1)	26 (36.6)	55 (32.5)	49 (43.0)	
Job Status (%)						0.14
Work on-line/from home	38 (40.9)	39 (47.6)	29 (40.8)	86 (50.9)	59 (51.8)	
Lost job due to COVID	11 (11.8)	5 (6.1)	4 (5.6)	7 (4.1)	10 (8.8)	
Lost wages due to COVID	14 (15.1)	10 (12.2)	7 (9.9)	30 (17.8)	19 (16.7)	
Essential Worker	9 (9.7)	9 (11.0)	14 (19.7)	17 (10.1)	6 (5.3)	
Not working, e.g., retired	21 (22.6)	19 (23.2)	17 (23.9)	29 (17.2)	20 (17.5)	
Age (%)						0.003 **
18–24	11 (11.8)	8 (9.8)	5 (7.0)	16 (9.5)	17 (14.9)	
25–34	24 (25.8)	26 (31.7)	13 (18.3)	57 (33.7)	49 (43.0)	
35–44	8 (8.6)	5 (6.1)	10 (14.1)	19 (11.2)	16 (14.0)	
45–54	16 (17.2)	8 (9.8)	8 (11.3)	22 (13.0)	12 (10.5)	
≥55	34 (36.6)	35 (42.7)	35 (49.3)	55 (32.5)	20 (17.5)	
Gender (%)						0.004 **
Male	26 (28.0)	28 (34.1)	20 (28.2)	49 (29.0)	14 (12.3)	
Female	67 (72.0)	54 (65.9)	51 (71.8)	120 (71.0)	100 (87.7)	
Non-binary	0 (0.0)	1 (1.2)	1 (1.2)	2 (1.2)	0 (0.0)	
Race (%)						0.004 **
White	82 (88.2)	75 (91.5)	57 (80.3)	144 (85.2)	83 (72.8)	
Non-white	11 (11.8)	7 (8.5)	14 (19.7)	25 (14.8)	31 (27.2)	
Urbanicity (%)						<0.001 ***
Large city	19 (20.4)	20 (24.4)	27 (38.0)	62 (36.7)	57 (50.0)	
Suburb	37 (39.8)	30 (36.6)	24 (33.8)	71 (42.0)	33 (28.9)	
Small town	22 (23.7)	19 (23.2)	16 (22.5)	26 (15.4)	22 (19.3)	
Rural	15 (16.1)	13 (15.9)	4 (5.6)	10 (5.9)	2 (1.8)	
Poverty (%)						0.17
Low	16 (17.2)	19 (23.2)	14 (19.7)	30 (17.8)	15 (13.2)	
Low-Medium	39 (41.9)	36 (43.9)	21 (29.6)	68 (40.2)	36 (31.6)	
Medium	26 (28.0)	15 (18.3)	20 (28.2)	49 (29.0)	38 (33.3)	
High	12 (12.9)	12 (14.6)	16 (22.5)	22 (13.0)	25 (21.9)	
Region (%)						0.21
Northeast	46 (49.5)	37 (45.1)	36 (50.7)	67 (39.6)	44 (38.6)	
Southeast	16 (17.2)	11 (13.4)	15 (21.1)	41 (24.3)	23 (20.2)	
Central	6 (6.5)	3 (3.7)	0 (0.0)	10 (5.9)	2 (1.8)	
Southwest	5 (5.4)	7 (8.5)	5 (7.0)	14 (8.3)	7 (6.1)	
West	20 (21.5)	24 (29.3)	15 (21.1)	37 (21.9)	38 (33.3)	
Household (%)						0.23
Live alone	12 (12.9)	11 (13.4)	12 (16.9)	35 (20.7)	28 (24.6)	
Live with spouse/partner	40 (43.0)	40 (48.8)	32 (45.1)	77 (45.6)	39 (34.2)	
Live with children < 18	17 (18.3)	11 (13.4)	7 (9.9)	17 (10.1)	11 (9.6)	
Live with roommate(s)	13 (14.0)	11 (13.4)	12 (16.9)	16 (9.5)	23 (20.2)	
Live with parents/family	11 (11.8)	9 (11.0)	8 (11.3)	24 (14.2)	13 (11.4)	

*p* value significance codes: 0 ‘***’ 0.001 ‘**’; ±95% CI = 95% Confidence Interval.

**Table 2:** Linear regression analysis illustrating effect of bivariable predictors and fully-adjusted multivariable model of nature deprivation under COVID-19 lockdown restrictions.

Outcome: Nature Deprivation (5-pt Scale)	Model 1: Bivariable			Model 2: Multivariable		
Variable	beta	95% CI	F-Test *p*	beta	95% CI	F-Test *p*
Nature Affinity						0.001 **
Low affinity = reference	---	---	0.34	---	---	
Medium affinity	0.27	(−0.12, 0.66)		0.46	(0.11, 0.80)	
High affinity	0.29	(−0.12, 0.69)		0.69	(0.32, 1.06)	
Pre-pandemic time in nature						0.60
Never or < monthly = reference	---	---	0.06	---	---	
1–2x/month	−0.64	(−1.67, 0.39)		−0.38	(−1.25, 0.48)	
1–2x/week	−0.8	(−1.80, 0.21)		−0.53	(−1.41, 0.34)	
3–4x/week	−0.62	(−1.61, 0.38)		−0.49	(−1.37, 0.40)	
Daily	−0.99	(−1.99, 0.01)		−0.61	(−1.50, 0.29)	
Time in nature during pandemic						<0.001 ***
Less time vs. pre-pandemic = reference	---	---	<0.001 ***	---	---	
Same time as pre-pandemic	−1.47	(−1.76, −1.19)		−0.98	(−1.28, −0.69)	
More time vs. pre-pandemic	−1.51	(−1.74, −1.28)		−1.07	(−1.32, −0.81)	
Municipal restriction on nature areas						<0.001 ***
Parks entirely shut to public = reference	---	---	<0.001 ***	---	---	
Foot/bike access only	−0.38	(−0.80, 0.04)		−0.47	(−0.83, −0.10)	
Parking spaces limited but not closed	−0.39	(−0.77, −0.00)		−0.21	(−0.55, 0.13)	
Full access to nature areas	−1.33	(−1.72, −0.95)		−0.81	(−1.17, −0.45)	
View from windows						0.18
Buildings, urban view = reference	---	---	<0.001***	---	---	
Street trees	−0.39	(−0.93, 0.16)		−0.25	(−0.72, 0.22)	
Lawns, some garden	−0.66	(−1.20, −0.11)		−0.05	(−0.54, 0.44)	
Woodlands	−1.31	(−1.87, −0.74)		−0.34	(−0.87, 0.19)	
Water views	−0.68	(−1.37, −0.01)		−0.03	(−0.64, 0.58)	
Pandemic mobility restrictions						<0.001 ***
Complete lockdown = reference	---	---	<0.001 ***	---	---	
Go outside but no nature contact	−0.39	(−0.97, 0.18)		−0.58	(−1.160, −0.0)	
Go outside, preferred N inaccessible	−0.56	(−1.02, −0.10)		−0.36	(−0.80, 0.07)	
No restriction: normal outdoor access	−1.79	(−2.24, −1.33)		−0.87	(−1.33, −0.41)	
Pets						0.01 *
Do not have pets = reference	---	---	0.001 **	---	---	
Have pets	−0.39	(−0.63, −0.15)		−0.27	(−0.46, −0.07)	
Age						0.17
18–24 = reference	---	---	0.003 **	---	---	
25–34	0.13	(−0.29, 0.55)		−0.12	(−0.46, 0.23)	
35–44	0.17	(−0.34, 0.68)		−0.06	(−0.47, 0.36)	
45–54	−0.26	(−0.75, 0.23)		−0.26	(−0.67, 0.15)	
≥55	−0.4	(−0.81, 0.02)		−0.36	(−0.71, −0.01)	
Gender						<0.001 ***
Male = reference	---	---	0.01 *	---	---	
Female	0.36	(0.09, 0.63)		0.4	(0.19, 0.63)	
Non-binary	0.27	(−1.13, 1.67)		−0.29	(−1.41, 0.84)	
Race						0.84
White, Non-Hispanic = reference	---	---	0.002 **	---	---	
Non-white	0.5	(0.18, 0.82)		−0.03	(−0.30, 0.25)	
Urbanicity						0.41
Large city = reference	---	---	<0.001 ***	---	---	
Suburb	−0.49	(−0.74, −0.19)		−0.1	(−0.36, 0.16)	
Small town	−0.57	(−0.90, −0.24)		−0.13	(−0.43, 0.16)	
Rural	−1.3	(−1.75, −0.85)		−0.37	(−0.80, 0.06)	
Poverty						0.93
Low = reference	---	---	0.15	---	---	
Low-Medium	0.03	(−0.31, 0.38)		−0.03	(−0.31, 0.25)	
Medium	0.3	(−0.07, 0.66)		0.02	(−0.29, 0.32)	
High	0.32	(−0.09, 0.73)		−0.08	(−0.44, 0.28)	
Region						0.07
Northeast = reference	---	---	0.24	---	---	
Southeast	0.3	(0.02, 0.63)		0.1	(−0.18, 0.37)	
Central	−0.16	(−0.79, 0.47)		−0.37	(−0.89, 0.14)	
Southwest	0.18	(−0.31, 0.66)		−0.02	(−0.41, 0.38)	
West	0.25	(−0.05, 0.55)		−0.3	(−0.57, −0.04)	

*p* value significance codes: 0 ‘***’ 0.001 ‘**’ 0.01 ‘*’; ±95% CI = 95% Confidence Interval.

**Table 3:** Linear regression analysis of bivariable model and multivariable model of individual flourishing adjusted for age and urbanicity under COVID-19 lockdown restrictions.

Outcome: Flourishing (90-Point Scale)	Model 1: Bivariable			Model 2: Multivariable		
Variable	beta	95% CI	F-Test *p*	beta	95% CI	F-Test *p*
Nature Affinity						0.06
Low affinity = reference	---	---	0.03 *	---	---	
Med affinity	0.33	(−2.99, 3.66)		−0.03	(−3.21, 3.15)	
High affinity	3.17	(−0.28, 6.62)		2.49	(−0.88, 5.87)	
Nature Deprivation						0.03 *
Strongly disagree feel nature deprived	---	---	<0.001 ***	---	---	
Disagree that feel nature deprived	1.01	(−2.54, 4.57)		0.47	(−2.91, 3.86)	
Neither agree nor disagree	0.42	(−3.28, 4.12)		−0.49	(−4.09, 3.11)	
Agree that feel nature deprived	−3.24	(−6.27, −0.21)		−2.72	(−5.68, 0.24)	
Strongly agree feel nature deprived	−5.52	(−8.80, −2.25)		−4.04	(−7.33, −0.74)	
Job Status						0.002 **
Work on-line/from home = reference	---	---		---	---	
Lost job due to COVID	−7.54	(−11.62, −3.45)	<0.001 ***	−6.75	(−10.78, −2.72)	
Lost wages due to COVID	−2.03	(−5.00, 0.95)		−3.42	(−6.36, −0.49)	
Essential worker	−0.51	(−3.96, 2.94)		−1.9	(−5.68, 1.61)	
Not working (retired, etc.)	4.9	(2.21, 7.58)		1.1	(−1.90, 3.92)	
Household						0.005 **
Live alone = reference	---	---	<0.001 ***	---	---	
Live with spouse/partner	6.2	(3.36, 9.03)		5.23	(2.52, 7.95)	
Live with children < 18	3.62	(−0.09, 7.35)		5.24	(1.22, 9.26)	
Live with roommate(s)	1.47	(−2.13, 5.06)		4.3	(0.60, 8.01)	
Live with parents/family	2.11	(−1.65, 5.86)		4.17	(0.19, 8.15)	
Age						<0.001 ***
18-24 = reference	---	---	<0.001 ***	---	---	
25–34	1.39	(−2.11, 4.88)		0.39	(−3.26, 4.04)	
35–44	1.38	(−2.88, 5.63)		0.55	(−4.24, 5.35)	
45–54	3.05	(−1.08, 7.17)		1.98	(−2.63, 6.58)	
≥55	9.19	(5.72, 12.66)		6.76	(2.63, 10.88)	
Gender						0.94
Male = reference	---	---	0.69	---	---	
Female	−0.47	(−2.85, 1.90)		−0.02	(−2.29, 2.25)	
Non-binary	−5.05	(−17.18, 7.07)		−4.49	(−15.91, 6.95)	
Race						0.19
White, Non-Hispanic = reference	---	---	0.75	---	---	
Non-white	−0.45	(−3.24, 2.34)		1.79	(−0.92, 4.50)	
Urbanicity						0.61
Large city = reference	---	---	0.13	---	---	
Suburb	1.47	(−0.97, 3.82)		−1.1	(−3.66, 1.47)	
Small town	2.79	(−0.12, 5.70)		0.44	(−2.47, 3.36)	
Rural	3.86	(−0.13, 7.86)		0.81	(−3.24, 4.85)	
Poverty						0.17
Low = reference	---	---	0.01 *	---	---	
Low_Medium	2.43	(−0.53, 5.39)		2.45	(−0.92, 5.33)	
Medium	−0.04	(−3.09, 3.17)		1.26	(−1.95, 4.48)	
High	−2.49	(−6.01, 1.04)		−0.59	(−4.32, 3.14)	
Region						0.52
Northeast = reference	---	---	0.25	---	---	
Southeast	−0.31	(−2.49, 3.11)		1.12	(−1.59, 3.82)	
Central	−4.99	(−10.43, 0.44)		−3.45	(−8.65, 1.75)	
Southwest	−0.85	(−5.02, 3.33)		−1.21	(−5.10, 2.86)	
West	−1.9	(−4.49, 0.69)		−0.14	(−2.74, 2.47)	

*p* value significance codes: 0 ‘***’ 0.001 ‘**’ 0.01 ‘*’; ±95% CI = 95% Confidence Interval. Model 2: Nature affinity, self-assessed nature deprivation, job status and household composition during COVID, pre-pandemic frequency in nature predicting flourishing, adjusted for age (in deciles) gender, race, urbanicity, area-level poverty, and region.

**Table 4:** Interaction of nature affinity, nature deprivation with sociodemographic variables to predict flourishing.

Interaction	Net Effect ^	*p* Value	95% CI
**Affinity**			
Age 18–24 = reference			
Medium affinity: Age 18–24	−2.78	0.47	(−10.27, 4.71)
High affinity: Age 18–24	−10.40	0.01 *	(−18.53, −2.28)
Age 25–34			
Medium affinity: Age 25–34	2.07	0.30	(−3.03, 7.17)
High affinity: Age 25–34	3.80	0.004 **	(−1.69, 9.28)
Age 35–44			
Medium affinity: Age 35–44	7.61	0.09	(−1.59, 16.81)
High affinity: Age 35–44	10.62	0.001 **	(0.72, 20.50)
Age 45–54			
Medium affinity: Age 45–54	2.71	0.40	(−8.01, 13.43)
High affinity: Age 45–54	11.43	0.001 **	(0.58, 22.23)
Age 55 >			
Medium affinity: Age >55	−3.39	0.90	(−9.70, 2.94)
High affinity: Age >55	−0.06	0.05.	(−6.42, 6.13)
Deprivation			
White Race = reference			
Don’t feel nature deprived: White	0.05	0.98	(−3.58, 3.47)
Neutral: White	−0.46	0.82	(−4.33, 3.42)
Feel nature deprived: White	−4.08	0.01 *	(−7.24, −0.93)
Feel strongly nature deprived: White	−6.03	<0.001 ***	(−9.60, −2.46)
Non-White Race			
Don’t feel nature deprived: Non-white	6.44	0.26	(−4.84, 17.63)
Neutral: Non-white	0.85	0.79	(−8.35, 10.96)
Feel nature deprived: Non-white	7.15	0.01 *	(2.63, 19.83)
Feel strongly nature deprived: Non-white	4.70	0.01 *	(2.25, 19.21)

*p* value significance codes: 0 ‘***’ 0.001 ‘**’ 0.01 ‘*’; ±95% CI = 95% Confidence Interval. ^ Footnote: net effect measures the role of affinity among individuals in the indicated age category.

## Data Availability

Data to support the study findings are available by request to corresponding author.

## Supplementary Materials

The following are available online at https://www.mdpi.com/1660-4601/18/4/1511/s1, Supplementary Materials S1: Qualtrics Nature Survey; Supplementary Materials S2: Harvard Flourishing Index.
