# Supplementary material for: The Relationship between Nature Deprivation and Individual Wellbeing across Urban Gradients under COVID-19
Source: Int J Environ Res Public Health. 2021 Feb 5;18(4):1511. doi: 10.3390/ijerph18041511 (PMC7915014; doi:10.3390/ijerph18041511)
Supplement: Supplementary file 1 [file ijerph-18-01511-s001.pdf]

HARVARD T.H. CHAN SCHOOL OF PUBLIC HEALTH

Appendix A: Qualtrics Survey

This questionnaire surveys your opinions about your current conditions affecting nature accessibility as a result of COVID-19 health restrictions and the relation of these factors to individual health and wellbeing. We ask you to answer objectively and accurately to the best of your recall. Survey responses are being collected by the Harvard Chan School of Public Health and will remain anonymous at the individual participant level. The entire survey will take approximately 10 minutes to complete.

For purposes of these questions, the term “outdoors” connotates time intentionally spent in or near nature: backyard, outdoor gardening, urban park, other urban/suburban greenspace, greenway for walking or biking, open woodlands, state or national park/forest/seashore, all forms of outdoor sport or recreation.

Nature access

1. Which of the following options would you say describes your relationship to nature: (1 represents no overlap between yourself and nature; 7 represents oneness with nature.

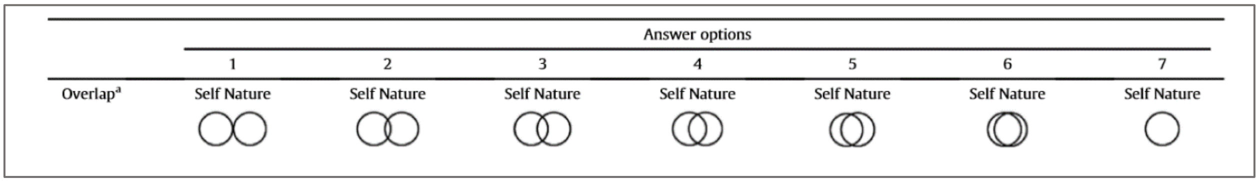

   a. 1
   b. 2
   c. 3
   d. 4
   e. 5
   f. 6
   g. 7

2. Describe your activity pattern in nature prior to any coronavirus restrictions in your area.
   a. I rarely or never spent time in nature
   b. I spent time in nature 1-2 times a month
   c. I spent time in nature 1-2 times a week
   d. I spent time in nature 3-5 times a week
   e. I spent time in nature daily

3. How would you describe the amount of time you now spend in or near nature under coronavirus restrictions as compared to the amount of time spent in or near nature prior to restrictions?
   a. Much less time
   b. Somewhat less time
   c. About the same
   d. Somewhat more time
   e. Much more time

4. Given restrictions on personal mobility within your city or state, can you freely access nature areas such as parks, conservation land, beaches, etc?
    a. No, nature areas have been entirely closed to the public
    b. Restricted access since parking lots have been closed to limit the number of people arriving by car
    c. Open access on foot or bicycle only
    d. Yes, full access with social distancing required

5. Have you ever thought, "I feel nature deprived," since coronavirus restrictions were imposed?
    a. Strongly disagree
    b. Disagree
    c. Neither agree nor disagree
    d. Agree
    e. Strongly agree

6. What scene predominates in the view from your window?
    a. Urban buildings, no nature
    b. Some nature, a few street trees
    c. Lawn, some garden elements
    d. Water (lake or pond, beachfront, commercial waterway)
    e. Woodland or other natural setting

7. How would you describe the seasonal stage of nature you currently see in your area?
    a. Dormant/still awaiting spring
    b. Desert conditions (if appropriate, specify desert 'season')
    c. Nascent/still emerging
    d. Fully flourishing

8. Does your indoor space include houseplants you care for?
    a. Y/N
    b. If Yes, how many

9. Do you have a pet at home?
    a. Y/N
    b. If yes, is it a pet which requires taking outdoors?

10. Please rank your in order of preference your top three activities:
    a. Being on-line or social media
    b. Outdoor recreation alone or with friends
    c. Spending time indoors with friends
    d. Watching TV (Netflix, etc)
    e. Indoor yoga, exercise (at home or going to gym)
    f. Reading, cooking or other personal interests
    g. Other _______________

11. What outdoor activity do you most often engage in outside of coronavirus restrictions? (write-in answer)

12. What have you found you enjoy the most during shelter-in-place restrictions? (write-in answer)

13. What do you miss the most during shelter-in-place restrictions? (write-in answer)

Current coronavirus circumstances.
The following questions relate to your current circumstances under coronavirus.

1. To what degree have your movements in the place you currently live affected by coronavirus?
   a. I am in complete lockdown, i.e., no time outdoors except for food and essentials
   b. I can go outdoors but do not come in contact with nature within that space
   c. I am able to go outdoors but my preferred outdoor recreational space is inaccessible
   d. I am able to access outdoor space in nature and normal physical activity outdoors without restriction

2. How long have you been sheltering-in-place due to coronavirus restrictions?
   a. 1-2 weeks
   b. 3-4 weeks
   c. More than 4 weeks
   d. Where I live is not affected by coronavirus restrictions
   e. I am considered an essential worker and leave home to do my job

3. Describe the effect of coronavirus on your ability to work.
   a. I have experienced some loss of work and income because of coronavirus shutdown
   b. I have lost my job because my workplace has been ordered closed due to coronavirus
   c. I am able to continue my regular work at home or online
   d. My work has been deemed essential during coronavirus and continues without interruption
   e. I do not work (retired or other)

4. Describe your personal mobility at present for accessing necessary goods and services.
   a. I own a car.
   b. I take public transit.
   c. I use ride-sharing services like Uber or Lyft.
   d. I walk or bike.
   e. I use delivery services or depend on drop-offs from family/friends.

HARVARD T.H. CHAN | SCHOOL OF PUBLIC HEALTH

Wellbeing questions

The following questions regard your sense of wellbeing over the past month.

1. I look for creative ways to alter difficult situations.
    a. Does not describe me at all
    b. Does not overall describe me
    c. Neutral
    d. Overall describes me
    e. Describes me very well

2. Regardless of what happens to me, I believe I can control my reaction to it.
    a. Does not describe me at all
    b. Does not overall describe me
    c. Neutral
    d. Overall describes me
    e. Describes me very well

3. I have so much in life to be thankful for.
    a. Strongly disagree
    b. Disagree
    c. Neither agree nor disagree
    d. Agree
    e. Strongly agree

4. I am feeling lonely due to coronavirus restrictions.
    a. Strongly disagree
    b. Disagree
    c. Neither agree nor disagree
    d. Agree
    e. Strongly agree

5. I cannot make sense of what is going on in the world.
    a. Strongly disagree
    b. Disagree
    c. Neither agree nor disagree
    d. Agree
    e. Strongly agree

6. I am grateful to a wide variety of people.
    a. Strongly disagree
    b. Disagree
    c. Neither agree nor disagree
    d. Agree
    e. Strongly agree

7. I feel close to others in my community.

a. Strongly disagree
b. Disagree
c. Neither agree nor disagree
d. Agree
e. Strongly agree

8. In the last month, how often have you felt that things were going your way?
   a. Never
   b. Almost never
   c. Sometimes
   d. Fairly often
   e. Very often

9. In the last month, how often have you felt confident about your ability to handle your personal problems?
   a. Never
   b. Almost never
   c. Sometimes
   d. Fairly often
   e. Very often

10. In the last month, how often have you felt difficulties were piling up so high that you could not overcome them?
   a. Never
   b. Almost never
   c. Sometimes
   d. Fairly often
   e. Very often

11. In general, how happy or unhappy do you usually feel?

Unhappy — Happy

0○ 1○ 2○ 3○ 4○ 5○ 6○ 7○ 8○ 9○ 10○

12. How would you rate your physical health over the last month?

Unhappy — Happy

0○ 1○ 2○ 3○ 4○ 5○ 6○ 7○ 8○ 9○ 10○

13. How would you rate your overall emotional health over the last month?

Unhappy — Happy

0○ 1○ 2○ 3○ 4○ 5○ 6○ 7○ 8○ 9○ 10○

14. Overall, to what extent do you feel the things you do in your life are worthwhile?

Unhappy — Happy

0○ 1○ 2○ 3○ 4○ 5○ 6○ 7○ 8○ 9○ 10○

15. I understand my purpose in life.

Unhappy — Happy

0◯ 1◯ 2◯ 3◯ 4◯ 5◯ 6◯ 7◯ 8◯ 9◯ 10◯

16. I always act to promote good in all circumstances, even in difficult and challenging situations.

Unhappy — Happy

0◯ 1◯ 2◯ 3◯ 4◯ 5◯ 6◯ 7◯ 8◯ 9◯ 10◯

17. I am content with my friendships and relationships.

Unhappy — Happy

0◯ 1◯ 2◯ 3◯ 4◯ 5◯ 6◯ 7◯ 8◯ 9◯ 10◯

18. How often do you worry about being able to meet normal monthly living expenses under coronavirus restrictions?

Unhappy — Happy

0◯ 1◯ 2◯ 3◯ 4◯ 5◯ 6◯ 7◯ 8◯ 9◯ 10◯

19. How often did you worry about safety, food, or housing prior to coronavirus restrictions?

Unhappy — Happy

0◯ 1◯ 2◯ 3◯ 4◯ 5◯ 6◯ 7◯ 8◯ 9◯ 10◯

Demographic questions:

Age group

18-24
25 - 34
35 - 44
45 - 54
55 - 64
65 - 74
75 - 84

Gender

Male
Female
Non-Binary

Ethnicity or Cultural Identification (check all that apply)

White or Caucasian
African-American or Black

Native American
East Asian
South Asian
Hispanic or LatinX
Middle Eastern
African
Other _______________

Where do you consider your place of residence to be? (check all that apply)

Large city or urban area
Suburban area
Small city or town
Rural area
East coast
South
Central
West coast

In which state are you are taking this survey? __________________

In which zip code are you are taking this survey? _________________

Current living situation (check all that apply)

I live alone
I live with my spouse or partner
I live with my parents/family
I have children under the age of 18 currently living with me
I share living space with roommates
I live in an apartment with central building access
I live in a stand-alone residence or building unit with direct outdoor access

How many children under age 18 live with you? ______________

HARVARD T.H. CHAN | SCHOOL OF PUBLIC HEALTH

Appendix B: Harvard Flourishing Index

The Human Flourishing Program has developed a measurement approach to human flourishing, based around five central domains: happiness and life satisfaction, mental and physical health, meaning and purpose, character and virtue, and close social relationships. Each of these is nearly universally desired, and each constitutes an end in and of itself.

The measure and its conceptual motivation were put forward in a 2017 paper, On the Promotion of Human Flourishing, by Professor VanderWeele, in the Proceedings of the National Academy of Sciences. The Program aims to encourage the measurement and tracking of these various aspects of flourishing in workplace, medical, educational, and governmental settings, and to better understand the determinants of flourishing, and policies to promote it. Together with the SHINE Program at the Harvard T.H. Chan School of Public Health, the measure has been used in collaboration with Levi Strauss & Co., Allegacy Credit Union, Owens Corning, several major airlines, and also a new multi-year research collaboration with Aetna, to expand and develop the measure yet further, and to track and promote well-being among employees.

The Program's flourishing measure is copyrighted under a Creative Commons License. However, it can be used without permission for non-commercial purposes if proper citation is given. The reference for the paper in which the measure was presented is:

> VanderWeele, T.J. (2017). On the promotion of human flourishing. Proceedings of the National Academy of Sciences, U.S.A., 31:8148-8156.

The "Flourish" measure consists of two questions or items from each of five domains: happiness and life satisfaction, mental and physical health, meaning and purpose, character and virtue, and close social relationships. The questions were selected primarily from among existing questions that had received some empirical validation and that are widely used in the well-being literature. Each of the questions is assessed on a scale of 0 – 10.

The "Flourish" measure is obtained by summing the scores from each of the ten questions and results a total score from of 0 – 100. The "Secure Flourish" measure also includes two additional questions on financial and material stability which may indicate the capacity to sustain flourishing into the future across the five principal domains; the "Secure Flourish" measure is obtained by summing the scores from the twelve questions in all six domains and results in a score from 0 – 120.

Flourishing Questions

Domain 1: Happiness and Life Satisfaction.

1. Overall, how satisfied are you with life as a whole these days?
0=Not Satisfied at All, 10=Completely Satisfied

2. In general, how happy or unhappy do you usually feel?
0=Extremely Unhappy, 10=Extremely Happy

Domain 2: Mental and Physical Health.

3. In general, how would you rate your physical health?
0=Poor, 10=Excellent

4. How would you rate your overall mental health?
0=Poor, 10=Excellent

Domain 3: Meaning and Purpose.

5. Overall, to what extent do you feel the things you do in your life are worthwhile?
0=Not at All Worthwhile, 10=Completely Worthwhile

6. I understand my purpose in life.
0=Strongly Disagree, 10=Strongly Agree

Domain 4: Character and Virtue.

7. I always act to promote good in all circumstances, even in difficult and challenging situations.
0=Not True of Me, 10=Completely True of Me

8. I am always able to give up some happiness now for greater happiness later.
0=Not True of Me, 10=Completely True of Me

Domain 5: Close Social Relationships.

9. I am content with my friendships and relationships.
0=Strongly Disagree, 10=Strongly Agree

10. My relationships are as satisfying as I would want them to be.
0=Strongly Disagree, 10=Strongly Agree

Domain 6: Financial and Material Stability.

11. How often do you worry about being able to meet normal monthly living expenses?
0=Worry All of the Time, 10=Do Not Ever Worry

12. How often do you worry about safety, food, or housing?
0=Worry All of the Time, 10=Do Not Ever Worry

For further information on the psychometric properties of the measures, see:
Węziak-Białowolska, D., McNeely, E., and VanderWeele, T.J. (2018). Flourish index and secure flourish index – development and validation. Available at SSRN: https://papers.ssrn.com/sol3/papers.cfm?abstract_id=3145336.
